# Response of the Gypsy Moth, *Lymantria dispar* to Transgenic Poplar, *Populus simonii* x *P. nigra*, Expressing Fusion Protein Gene of the Spider Insecticidal Peptide and *Bt*-toxin C-peptide

**DOI:** 10.1673/031.010.20001

**Published:** 2010-11-17

**Authors:** Chuan-Wang Cao, Gui-Feng Liu, Zhi-Ying Wang, Shan-Chun Yan, Ling Ma, Chuan-Ping Yang

**Affiliations:** ^1^ Key Laboratory of Forest Tree Genetic Improvement and Biotechnology (Northeast Forestry University), Ministry of Education, Harbin 150040, China; ^2^ Department of Forestry Protection, Northeast Forestry University 150040, China

**Keywords:** biological control, moth, developmental effect, genetic engineering

## Abstract

The response of the Asian gypsy moth *Lymantria dispar* (L.) (Lepidoptera: Lymantriidae) to a fusion gene consisting of the spider, *Atrax robustus* Simon (Araneae: Hexanthelidae) ω?-ACTX-Ar1 sequence coding for an ω?-atracotoxin and a sequence coding for the *Bt*-toxin C-peptide, expressed in transgenic poplar *Populus simonii* x *P. nigra* L. (Malphigiales: Salicaceae) was investigated. Individual performance, feeding selection, midgut proteinase activity and nutrition utilization were monitored. The growth and development of *L. dispar* were significantly affected by continually feeding on the transgenic poplar, with the larval instars displaying significantly shorter developmental times than those fed on nontransgenic poplar, but pupation was delayed. Mortality was higher in populations fed transgenic poplar leaves, than for larvae fed nontransgenic poplar leaves. The cumulative mortality during all stages of larvae fed transgenic leaves was 92% compared to 16.7% of larvae on nontransgenic leaves. The highest mortality observed was 71.7% in the last larval instar stage. A two-choice test showed that fifth-instar larvae preferred to feed on nontransgenic leaves at a ratio of 1:1.4. Feeding on transgenic leaves had highly significant negative effects on relative growth of larvae, and the efficiency of conversion of ingested and digested food. Activity of major midgut proteinases was measured using substrates TAME and BTEE showed significant increases in tryptase and chymotrypsinlike activity (9.2- and 9.0-fold, respectively) in fifth-instar larvae fed on transgenic leaves over control. These results suggest transgenic poplar is resistant to *L. dispar*, and the mature *L. dispar* may be weakened by the transgenic plants due to *Bt* protoxins activated by elevated major midgut proteinase activity. The new transgenic poplar expressing fusion protein genes of *Bt* and a new spider insecticidal peptide are good candidates for managing gypsy moth.

## Introduction

Insect control remains one of the major problems of modern agriculture despite the extensive use of chemical pesticides (Wu et al. 1997). A range of insecticides have been used including organophosphates, carbamates and pyrethroids (Maxwell 1992; Wheelock et al. 2005). However, most insect pests have evolved resistance to these some insecticides at a rate faster than new chemicals can be developed and registered (Metcalf 1986; Wu et al. 1997). A possible alternative solution to the problem posed by chemical pesticides is biological control or genetically modified plants. Many compounds are investigated for their possible use as bioinsecticidal agents for the control of phytophagous pests or vectors of new or re-emerging diseases (Tabashnik et al. 2005; Bates et al. 2005). To date, the most common organism used as a bioinsecticide is the bacteria, *Bacillus thuringiensis* (*Bt*), which forms crystals containing insecticidal proteins (Schnepf et al. 1998). After oral uptake by a susceptible species, the *Bt* toxin binding to specific receptors in the mid-gut epithelium of the larvae causes cell lysis leading to the death of the insect (Whalon and Wingerd 2003). Transgenic plants expressing insecticidal proteins from the *Bt* have become a major insecticide during last decade (Ferry et al. 2006). However, a recent study demonstrated transgenic plants expressing *Bt* genes have developed insect resistance (Tabashnik et al. 2008). To avoid or delay insect resistance, one option is to express various *Bt* genes in plants (Maqbool et al. 2001; Kurtz et al. 2007) or combine a variety of toxins to *Bt* proteins producing different toxic effects (Zhao et al. 2003; Naimov et al. 2003; Mehlo et al. 2005). Zhao et al. (2003) reported the resistance of the diamondback moths to pyramided two-gene, Cry 1 Ac and Cry 1 C, plants was significantly delayed as compared with resistance to single-gene plants. Another potentially useful toxin in this respect is spider venoms that are a complex mixture of molecules and peptides that have evolved specifically to kill insects, thus having a great potential for biotechnology application. Hernáández-Campuzano et al. (2009) demonstrated that the expression of a peptide toxin Magi 6 produced by the spider, *Macrothele gigas* in transgenic plants conferred resistance to attack by *Spodoptera frugiperda*.

The insecticidal peptide from the spider, *Atrax robustus* Simon (Araneae: Hexanthelidae) was synthesized and confirmed resistance to some agricultural pests by National Laboratory of Protein Engineering and Plant Genetic Engineering (NLPE&PGE), Beijing, China in cooperation with Deakin Company (Jiang et al. 1995, 1996a). Jiang et al. (1996a, b) reported that the mortality of *Heliothis armigera* fed artificial diet containing expressed protein product of *Escherichia coli* expressing spider insecticidal peptide was 86.7%, and three transgenic tobacco lines expressing spider insecticidal gene was 30∼?45%, respectively (Jiang et al. 1996a; Jiang et al. 1996b). They further constructed a vector containing fusion genes of spider insecticidal peptide from *A. robustus* and *Bt*-toxin C-peptide and transformed this vector into cotton to study resistance of cotton insects (Zheng et al. 2002). In assessing potential effects of the spider insecticidal peptide and *Bt*-toxin C-peptide on Lepidoptera, we also successfully transformed the fusion protein gene of the spider insecticidal peptide and *Bt*-toxin C-peptide into poplar, *Populus simonii* x *P. nigra* and *Betula platyphylla*, and found that the transformed poplar was resistant to *Clostera anachoreta* and *Lymantria dispar* (Fan et al. 2006; Wang et al. 2007). In order to extend observations and explore the possible use of a fused gene of *Bt* and spider insecticidal peptide as a bioinsecticide, we here examine the insecticidal effects of transgenic *Populus simonii* x *P. nigra* L. (Malphigiales: Salicaceae) expressing the spider insecticidal peptide and *Bt*-toxin C-peptides on the gypsy moth *Lymantria dispar* (L.) (Lepidoptera: Lymantriidae).

The gypsy moth, *L. dispar* is a key lepidopteran species occurring in forests in many countries. It is estimated that worldwide at least 500 plants are hosts to *L. dispar*, predominantly poplar, oak and birch (Lazareviae et al. 1998). Therefore, *L. dispar* was selected to quantify the insecticidal effect of transgenic poplar with the fusion protein gene of the spider insecticidal peptide and *Bt-*toxin C-peptide in controlled laboratory experiments. Larvae of *L. dispar* were fed with nontransgenic and transgenic poplars and the resulting effects were analyzed with respect to individual performance, feeding selection, nutrition utilization and midgut proteinase activity of the larvae.

## Materials and methods

### Plasmid construction and plants

The gene encoding the 37 amino acid spider insecticidal peptide from *A. robustus* (ω?-ACTX-Ar1) was synthesized by National Laboratory of Protein Engineering and Plant Genetic Engineering (NLPE&PGE), Beijing, China in cooperation with Deakin Company according to plant codon preference (Jiang et al. 1995, 1996a). The fused gene was comprised of the toxin gene from *A. robustus* (ω?-ACTX-Ar1) (Wang et al. 1999) and the C terminal of the Cry I A (b) gene from *Bt*, and the binary expression vector of the fused gene, pYHY, was kindly provided by NLPE&PGE. *Agrobarcterium tumefaciens* strain LBA4404 containing binary vector plasmid pYHY was used to transform the fused gene into poplar *Populus simonii* x *P. nigra*. Three transgenic poplar lines named TT1, TT2 and TT3 were obtained and confirmed by PCR and Southern analyses (Jiang et al. 2004). In this study, the transgenic poplar line TT2 was selected in resistance experiments to *L. dispar* according to preliminary bioassays (data not shown). The nontransgenic and transgenic TT2 poplars were clonally propagated in tissue culture, transferred to soil and grown in a mixture of peat and sand (2:1 v/v) in a greenhouse with 24°° C day and 12°° C night temperature conditions. No chemical sprays were applied to the poplars before or during the experiments.

### Lymantria dispar

*L. dispar* eggs were collected in a mixed forest with predominantly Asian white birch trees (*Betula platyphylla*) from the Forestry Centre in Northeast Forestry University in February of 2008. The egg masses were kept at 4°° C from February to April, when they were ready hatch. The hatched caterpillars from the egg masses were divided into two groups: one group was reared on nontransgenic poplar (*Populus simonii* x *P. nigra*) and the other on transgenic poplar (*Populus simonii* x *P. nigra* TT2 line). The insects were reared at 25°° C and 14:10 L:D. Transparent plastic 250 ml bottles were used to rear larvae. Five first instar or 1∼?2 later instars were reared per bottle, while the pupae were reared individually. The humidity in the plastic bottles was maintained by botanical sponges soaked with water, which also kept the leaves fresh. Moths were fed with a 10% honey solution and mated in a 40 x 30 x 20 cm cage with a removable mesh cloth top for egg collection.

### Insect Feeding Trials and Individual Performance Measurement

The performance of individual insects reared on the nontransgenic and transgenic poplars was determined by measuring the following traits: developmental time of larval instars (LDT), development time from *L. dispar* hatching to adult emergence (DT), pupal duration (PD), pupal mass on the second day of development (PM), adult longevity (L), sex ratio (♀?/♂?), total number of eggs laid by female adult and cumulative mortality of larval instar. The total survival and development from *L. dispar* hatching to adult emergence was determined daily. The cumulative mortality (%) = 100 x(X_1_ + X_2_ + ……+X_6_) / N, where X_1_∼?X_6_ is the number of dead larvae at the 1∼?6 th larval instar, respectively, and N is the total number of 1∼?6th larval instar *L. dispar*. 12 and 20 replicates were made for nontransgenic and transgenic leaves treatments, respectively, with 10 individuals per replicate in this experiment.

### Larval Feeding Preference in Choice Test

Choice tests were designed to determine the feeding preference for nontransgenic and transgenic poplars by fifth instars. The choices consisted of newly expanded leaf discs (15 mm in diameter) of two plant species and arranged in ABAB sequence around the circumference of the Petri dishes (12 cm in diameter) containing moist filter paper on the floor. One freshly molted fifth instar was placed in the center of each Petri dish and kept in dark for 2 h. The area of each disc consumed was measured using transparency film (PP 2910, 3M Co., www.3M.com) with a 1-mm2 grid and recorded. The percentage of total consumption of each plant (choice index) was calculated for each larva as a measure of its feeding preference, as follows: Percent total consumption of nontransgenic poplar = (consumption of nontransgenic poplar) / (consumption of nontransgenic + transgenic poplar) and percent total of transgenic poplar = (consumption of transgenic poplar) / (consumption of nontransgenic + transgenic poplar).

Thirty *L. dispar* larvae were used in this experiment.

### Midgut Proteinase Assays

Larvae of *L. dispar* used for midgut proteinase assays were continually raised either on selected transgenic poplar TT2 lines or on control nontransgenic poplar. For each treatment, three replicates with 4 individuals per replicate were set up. The fifth instars randomly selected from each treatment were weighed and their midguts were dissected out in an ice-cold 0.15 M KC1 solution. The dissected midguts were blotted dry on tissue paper and stored at -80°° C until required.

Enzyme extracts were prepared by grinding thawed midgut samples in one volume of extraction buffer (0.15 M NaC1, 5% (w/v) polyvinyl-polypyrrolidone, and 0.03 M sodium diethyldithiocarbamate) (Jongsma et al. 1995). The extract was centrifuged at 4°°C for 20 min at 11,000 ×× *g* and the supernatant used for enzyme activity assays. Protein content was determined by the Bradford method (1976) using bovine serum albumin as the standard.

Substrates used for trypsin and chymotrypsin assays were 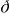-toluenesulfol-L-arginine methyl ester (TAME) and *N*-benzoyl-L-tyrosine ethyl ester (BTEE) respectively, and the assay procedures were essentially carried out as described in Wu et al. 1997. Trypsin assays were performed in quartz cuvettes of 3.5 ml capacity by mixing 1.5 ml 2 mM TAME, 1.4 ml assay buffer (0.04 M Tris, 0.01 M CaCl2, pH 8.1) and 100 μ? 1 diluted gut extract containing 20 μ? g of gut protein. The absorbance at 247 nm was recorded for 5 min at 25°° C. For chymotrypsin, the assays were carried out by mixing 1.5 ml 1 mM BTEE, 1.4 ml assay buffer (0.1 M Tris, 0.1 M CaCl_2_, pH 7.8), 100 μ? 1 diluted gut extract containing 20 μ?g protein, and recording the absorbance at 256 nm for 5 min at 25°° C. The proteinase activity is expressed as Δ?OD per minute per mg gut protein.

### Nutrition Utilization

The assay procedures were carried out according to the methods of Waldbauer 1964. Fifth instars (<10 hr old) were randomly selected to be tested. Larvae were allowed to feed for 24 h on each of the two nontransgenic and transgenic poplars in sufficient quantities in Petri dishes (12 mm in diameter) with moist filter paper. Initial dry weight of each larva was estimated by setting aside aliquots at the beginning of the experiment and a fresh/dry conversion ratio was obtained. The following growth indices were calculated as follows:

Relative consumption rate [RCR = dry weight of food ingested / (days of period X mean weight of larva during the period)]

Relative growth rate [RGR = dry weight gained of larva during feeding period / (days in feeding period X mean dry weight of larva during feeding period)]

Eficiency of conversion of ingested food [ECI = 100 X dry weight gained / dry weight food ingested]

Efficiency of conversion of digested food [ECD = 100 X dry weight gained / (dry weight food ingested —— dry weight feces)]

Approximate digestibility [AD = 100 X (dry weight food ingested —— dry weight feces) / dry weight food ingested].

Twenty *L. dispar* larvae were used in this test.

## Data analyses

Data collected were subjected to analyses of variance (ANOVA) for differences in treatments. Independent samples *t*-test was used for data analyses in individual performances. A value of P<0.05 was considered statistically significant. All data were analyzed using SPSS software package version 11.5 for Windows (SPSS Inc., www.spss.com).

## Results

### Individual Performance

**Figure 1.**
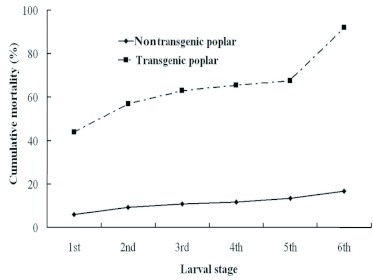
Cumulative mortality (% dead insects) at each larval instar for *L. dispar* fed on the nontransgenic or on transgenic poplar. Nontransgenic poplar: black line with diamond,Transgenic poplar: dotted line with square. Initial individuals based on 12 replicates of 10 individuals for nontransgenic and 20 replicates of 10 individuals for transgenic poplars. High quality figures are available online.

From the data presented in Table 1 and 2 and Figure 1, it is evident that the transgenic plant decreased the individual performance. Insecticidal genes in transgenic poplar leaves negatively affected the growth and development of *L. dispar* during most developmental stages compared to the control larvae fed on nontransgenic leaves (Tables 1 and 2). The developmental time of l–5th instars fed on transgenic leaves was decreased compared to insects fed on nontransgenic leaves (Table 1). However, the surviving 6th instars fed on transgenic poplar leaves delayed pupation compared to the control 6th instars, indicating that it may take some time to adapt to the toxic transgenic poplar. Similarly, the pupal duration of surviving larvae grown on transgenic leaves was significantly longer than those fed control leaves (F=6.19, P<0.05) (Table 2). For larvae fed on transgenic poplar, the longevity of adults increased 2.1 days. The sex ratio (♀?/♂?) of of adults under control compared to 0.1:1 for adults under transgenic treatments. The total number of eggs laid in the control and transgenic treatments were 2,516 and 95, respectively.

**Table 1.**
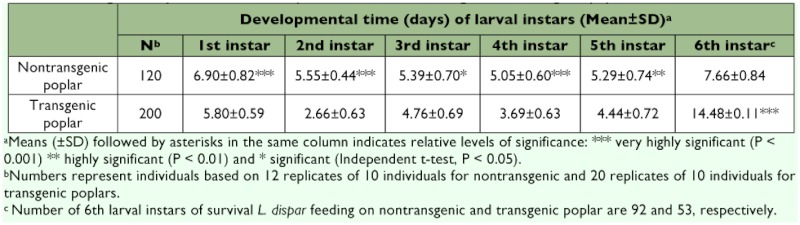
Average developmental time of *L. dispar* larvae fed on nontransgenic and transgenic poplar.

**Table 2.**
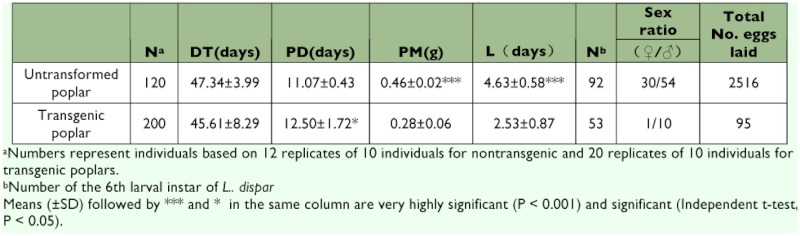
Pre-adult development time (DT), pupal duration (PD), pupal mass (PM), adult longevity (L), sex ratio (♀?/♂?) and total No. eggs

Many larvae were dead at most developmental stages when fed on transgenic poplar leaves (Figure 1). Mortality increased at developmental stages in both control and transgenic fed populations, but particularly for larvae fed transgenic poplar leaves (Figure 1). Ingestion of transgenic plants resulted in 92.0% cumulative mortality compared to only 16.7% of larvae fed nontransgenic poplar leaves. The greatest mortality observed was 71.7% of larvae fed transgenic poplar leaves compared to only 2.2% of larvae fed nontransgenic poplar leaves in the 6th larval instar stage.

### Feeding Selection Trials

**Figure 2.**
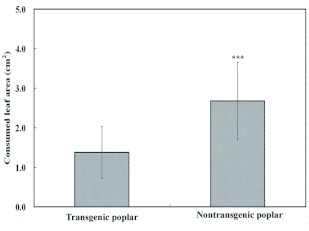
Area consumed of transgenic poplar leaf discs and nontransgenic poplar leaf discs by the fifth instar of *L. dispar* in 2 h choice experiments.****** indicates very high significance between nontransgenic and transgenic poplar (n = 30, P < 0.001). High quality figures are available online.

In the two-choice test, our results showed that fifth instar *L. dispar* preferred to feed on normal poplar (Figure 2), as evidenced in the significant differences in leaf consumption for these two treatments. The choice index of *L. dispar* was 0.7 ±± 0.1 to nontransgenic poplar by calculating the ratio between area of nontransgenic leaf discs eaten and total area of both nontransgenic and transgenic leaf discs eaten.

**Table 3.**
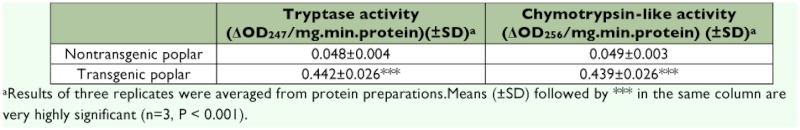
Effects of transgenic poplars on the midgut proteinase activity of the 5th instars of *L. dispar*.

### Midgut Proteinase Assays

In order to understand the nature of *L. dispar* response to transgenic leaves, the proteinase activity in the midguts of larvae fed on nontransgenic and transgenic leaves was measured in the fifth instar larvae of the feeding trial described above using TAME and BTEE as substrates. The tryptase and chymotrypsin-like activities were significantly higher (9.2- and 9.0-fold) in larvae feeding on transgenic than those feeding on nontransgenic leaves (Table 3).

### Nutrition Utilization

The nutrition indices of fifth instar of *L. dispar* on nontransgenic and transgenic poplars are shown in Table 4. Comparing each nutrition utilization index of *L. dispar* feeding on nontransgenic and transgenic poplars, it was found that the relative growth ratio (F=115.97, P<0.001), efficiency of conversion of ingested food (F=162.06, P<0.001) and efficiency of conversion of digested food (F=1566.60, P<0.001) of *L. dispar* were significantly greater on the nontransgenic poplar than on the transgenic poplar; however, relative consumption rate and approximate digestibility of *L. dispar* feeding on nontransgenic poplar is significantly lower than on transgenic poplar (F=32.44, P<0.001 and F=10.62, P<0.01, respectively).

## Discussion

Transgenic plants producing *Bt* toxin to control their key pests provide an attractive alternative to conventional insecticide sprays (Scott and Wilkinson 1998). *Bt* toxin acted on insect mid-gut epithelium causing physiology variation resulting in death (Whalon and Wingerd 2003). For example, the development of transgenic cotton varieties with the *Bt* gene has been one of the most successful applications of biotechnology research in China (Wu et al. 2003). Moreover, with heavy use of *Bt* transgenic plants such as *Bt* cotton, resistance developed in some insects (McGaughey 1985; Huang et al. 1999; Ferréé and Van Rie 2002). In order to overcome the resistance problem, researchers have begun to develop new transgenic plants expressing spider insecticidal peptide gene or fusion protein genes (Jiang et al. 1996b; Zheng et al. 2002). Spider venoms contain a complex natural library of polypeptide components, and many toxic peptides isolated from venomous spiders are known to inhibit voltage-sensitive ion channels with high selectivity of channel subtypes (Nicholson 2007). Transgenic tobacco expressed spider insecticidal peptide gene from *A. robustus* showed stronger toxicity to cotton bollworm than nontransgenic tobacco (Jiang et al. 1996b). The purpose of this study was to investigate the potential toxicity of transgenic hybrid poplar [*Populus simonii* x *P. nigra*] expressing a fusion protein gene composed of the sequence ω?-ACTX-Ar1 that codes for a spider insecticidal peptide, uω?-atracotoxin (Wang et al. 1999), and the *Bt*-toxin C-peptide at all of the developmental stages of *L. dispar*.

**Table 4.**
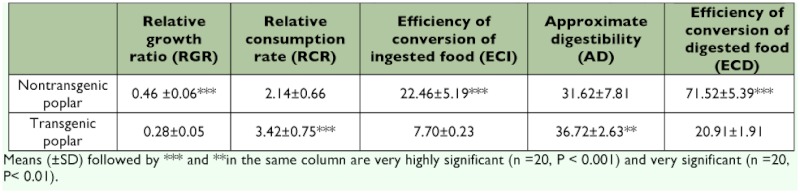
Nutrition indices of 5th instars of *L. dispar* feeding on leaves of nontransgenic and transgenic poplar.

The results showed that transgenic poplar expressed fusion protein gene of the spider insecticidal peptide and *Bt*-toxin C-peptide were lethal to all developmental stages for *L. dispar* (from first instar to adults). The transgenic poplar affects the developmental time, pupa weight, adult longevity, adult sex ratio and adult oviposition. The surviving *L. dispar* larvae may respond to inhibition of consumption and concurrent feeding on transgenic plants by maturing faster. Reserves acquired during the larval stages can be important for the moths’’ reproductive output as adults (Oberhauser 1997), and a curtailed larval stage may compensate for food shortage experienced early in larval life. Feeding on the transgenic *Populus simonii* x *P. nigra* also inhibited ecdyses of *Clostera anachoreta* larvae resulting in increasing larval developmental time (Fan et al. 2006). In the two-choice feeding test, the results showed that the fifth instars preferred to feed on the nontransgenic poplar (choice index=0.7). This may be associated with lower nutrient utilization of transgenic poplar and higher midgut proteinase activating *Bt* protoxins. The efficiency of conversion of digested food (ECD) and efficiency of conversion of ingested food (ECI) indicate that the nutritional value of transgenic poplar, and the level of nutrient intake of *L. dispar* decreased the proportion of food ingested that is required for maintenance, and were thus unavailable for growth; however, the relative consumption rate (RCR) and approximate digestibility (AD) of *L. dispar* were higher than on untransgenic nontransgenic poplar. Interestingly, tryptase activity and chymotrypsin-like activity in fifth instar *L. dispar* fed transgenic poplar was significantly increased compared to those fed nontransgenic poplar (F=1108.83, P<0.001 and F=615.88, P<0.001, respectively), which may be associated with increased activation of *Bt* protoxins.

**Figure 3.**
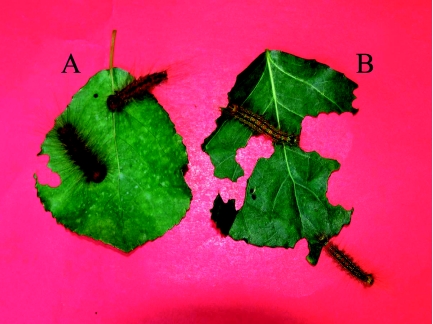
Photo showing the dead (A) and healthy (B) sixth stage instar larvae of *L. dispar* feeding on transgenic and nontransgenic poplars, respectively.The dead body on the transgenic poplar leaves became shriveled with foul odor, while the larvae on nontransgenic poplar leaves were healthy and vigorous. Therefore, the transgenic plant was protected from damage compared to the control plant. High quality figures are available online.

After ingestion, *Bt* protoxins are activated mainly by trypsins and/or chymotrypsins of insect gut proteinases. The major functions of the gut proteinases include hydrolyzation of ingested protein into peptides during the initial stages of protein catabolism. Serine proteinases are common luminal enzymes in the midguts of many Lepidoptera species (Applebaum 1985; Terra and Ferriera 1994). Some studies confirmed serine proteinases in the midgut activate protoxins, thereby mediating *Bt* toxicity (Milne and Kaplan 1993; Martíínez-Ramíírez and Real 1996), while they also may play a concurrent role in *Bt* resistance caused by degradation of toxins by gut proteinases in resistant strains (Forcada et al. 1996; Keller et al. 1996). Although the elevated gut proteinases may degrade *Bt* toxin, we believe the accumulation of toxic proteins in transgenic poplar resulted in high mortality of the sixth instars and low adult oviposition of *L. dispar*. The last instar dead larvae remains were rancid and soft (Figure 3). This result may be explained by accumulated toxicity. The results of this study suggest that the increased gut proteinase in fifth instar *L. dispar* feeding on transgenic poplars may not excessively degrade but rather activate *Bt* protoxins. Previous comparative studies focused on the effects of *Bt* toxin on young larvae (Olsen and Daly 2000; Peacock et al. 1998), or on a single developmental stage (Cheng et al. 1998). However, given the significant level of gene flow within and between standings of poplar (Géénissel et al. 2000), we believe that the *Bt* toxicity level must also be assessed for wandering mature larvae and adults. In addition, our study further showed that it is also important to estimate resistance in later life stages and evaluate long-term effects.

Based on the above observations, it would be expected that transgenic poplar *Populus simonii* x *P. nigra* expressing the fusion protein gene of the spider insecticidal peptide and *Bt*-toxin C-peptide would enhance insect resistance in poplars. These new transgenic poplars are good candidates to test against current strategies for delaying the appearance of pest resistance. To gain further insight into the bio-safety of the transgenic poplar, additional field and laboratory experiments will be carried out.
